# Emerging novel methodologies to understand and strategically target long-lived plasma cells in vaccine design to induce durable immunity

**DOI:** 10.3389/fimmu.2025.1680375

**Published:** 2026-02-06

**Authors:** Gina Cusimano, Ryan P. Staupe, Nicole L. Sullivan

**Affiliations:** Merck & Co., Inc., Infectious Diseases and Vaccines, Rahway, NJ, United States

**Keywords:** long-lived plasma cells, vaccines, durability, ELISpot, Ig Trap, Nanovials, fluorescence-activated cell sorting, single cell RNA sequencing

## Abstract

Long-lived plasma cells (LLPCs) are a subset of antibody secreting cells (ASCs) that reside within lymphoid tissues, including the bone marrow (BM) and gut associated lymphoid tissues (GALT), and can secrete antigen-specific antibodies for up to decades or longer. Due to these traits, LLPCs serve as a crucial mediator for durable protective immunity. The signals needed to control the differentiation of LLPCs from naïve B cells, however, are not well understood. Accordingly, it remains a challenge to design vaccines that specifically drive LLPC generation and subsequent antibody durability. In this review, we discuss LLPC generation and heterogeneity following vaccination, vaccine design decisions known to impact immunological memory, and how novel emerging technologies can be used to inform on LLPC biology to enable LLPC targeting vaccine design.

## Introduction

Vaccines are one of the most effective tools for maintaining global public health by controlling disease and preventing infection (1). Vaccination induces humoral memory by driving germinal center (GC) reactions that generate antigen-specific memory B cells and long-lived plasma cells (LLPCs) (2). LLPCs reside within lymphoid tissues, such as the bone marrow (BM) and gut associated lymphoid tissues (GALT), where they can secrete high affinity, neutralizing antibodies for up to decades or longer (3, 4). Neutralizing antibodies have traditionally served as the primary correlate of protection against many pathogens, making LLPCs a key component of durable immunity (5). While induction of durably-maintained antibody responses, including neutralizing antibodies, is a primary goal in vaccine development (5, 6), the underlying molecular mechanisms that drive antibody durability are poorly understood. Without a general understanding of how to drive strong LLPC responses, strategic vaccine design can be a challenge.

Vaccine design encompasses several modifiable parameters that can impact immunological memory. These include, but are not limited, to vaccine platform (7), adjuvant (8), antigen valency (9), duration of antigen bioavailability (10), and route of administration (11). Long-term serology data demonstrate that different vaccines induce varying LLPC responses (4, 12). For example, virus-like particle vaccines such as Gardasil^®^9 against human papillomavirus (13–16) and live attenuated viral vaccines against measles, mumps, and rubella (4, 17) induce stable and durable protective antibody. In contrast, mRNA-LNP vaccines against severe acute respiratory syndrome coronavirus 2 (SARS-CoV-2) (17–19) and bacterial toxoid vaccines such as diphtheria, tetanus, and pertussis (DTaP and Tdap) (4, 20, 21) generate antibody titers that rapidly wane after vaccination. It remains unclear what parameters or combination of parameters within vaccine design are needed to target and drive LLPC responses to promote durable protective immunity (6).

Studying LLPCs comes with many biological, as well as technical challenges, which can impede the understanding of vaccine design that can drive these important responses. A substantial barrier to studying LLPCs is rarity and sampling hurdles, particularly in humans. LLPCs represent less than 1% of BM cellularity and due to the invasive nature of BM aspirates, limited studies that evaluate LLPCs induced by vaccination in humans have been conducted (3, 22, 23). Studying LLPCs has additional challenges as LLPCs undergo rapid ex vivo apoptosis making *in vitro* evaluation of these cells difficult (24). In addition, heterogeneity within the plasma cell (PC) compartment makes defining a distinctive phenotype of LLPCs challenging (25). Technological limitations have traditionally prevented genomic characterization at a single-cell level of antigen-specific LLPCs. Novel emerging technologies (26, 27) and techniques (28–31) can enable such characterization, which will be critical to understand molecular signatures of LLPCs that can then be targeted by vaccine design.

In this review we will discuss the current understandings of how LLPCs are generated and factors impacting their fate decision making. We will review vaccine design decisions with known impacts on immunological memory formation, heterogeneity of LLPCs developed in response to vaccination, and challenges of studying LLPCs. Finally, we will provide an overview of emerging technologies and techniques that can enable a deeper understanding of LLPC biology needed to fuel vaccine design that can target LLPCs.

## Vaccine driven germinal center reactions give rise to memory B cells and LLPCs

Germinal centers (GCs) are dynamic, microanatomical sites that form within the follicles of secondary lymphoid organs in response to either infection or vaccination (32, 33). Focusing on GC formation following vaccination, vaccine antigen is phagocytosed by antigen-presenting cells (APCs), including dendritic cells (DCs) which process and present vaccine antigen onto MHC class I and MHC class II molecules. Once DCs are activated by innate cues, such as cytokines, DCs increase the expression of costimulatory molecules (i.e. CD80 and CD86) and MHC molecules. This allows for efficient antigen presentation by DCs to their cognate naïve B and T cells, leading to B and T cell activation (34).

Activated naïve B cells can remain extrafollicularly where they can proliferate and differentiate into early memory B cells (MBC) and short-lived plasmablasts that generally express immunoglobulin M (IgM) with limited B cell receptor (BCR) mutations resulting in limited affinity (32, 35). These early IgM^+^ memory B cells can participate in secondary responses and IgM^+^ short-lived ASCs serve as a rapid source of antibody with low-mid affinity (32, 36).

Alternatively, activated B cells can enter the follicle and migrate to the T:B cell border where they can interact with helper T cells known as T follicular helper (TFH) cells (33, 37). Interaction with TFH promotes B cell entry into the GC, where they can migrate between two distinctive GC zones, the dark zone (DZ) and light zone (LZ) (33). These GC B cells that migrate into the DZ undergo rapid proliferation and somatic hypermutation resulting in a population of GC B cells with mutated BCRs (32, 38). These BCRs that have undergone somatic hypermutation have varying ability to bind to antigen resulting in a range of antigen affinities. Migration from the DZ to the LZ allows these GC B cells to interact with both follicular dendritic cells (FDCs) which have captured cognate vaccine antigen and TFH cells to drive affinity selection of newly mutated BCRs. Within the LZ, GC B cells can also interact with TFH cells which provide critical signals for their continued survival and proliferation (33, 37). This cycling of GC B cells between proliferation and somatic hypermutation in the DZ and antigen driven selection in the LZ leads to a population of class-switched high affinity GC B cell clones that can further differentiate into MBC and LLPCs (32, 39).

Unlike MBC, LLPCs require specialized lymphoid tissue niches such as BM for survival (3, 24, 40). How LLPC precursors migrate from lymphoid tissues into the BM remains largely unknown (6), with the exception of the transcription factor c-Myb being required for LLPC responsiveness to chemokine gradients needed to enter the BM (41). LLPCs are a unique cell type that undergoes drastic transcriptional, morphological, and metabolic reconfiguring to produce copious amounts of immunoglobulin while remaining non-proliferative (24, 36, 42). Immunoglobulin made by LLPCs can directly neutralize pathogens or mediate interaction with the innate immune system via Fc-mediated functions such as antibody dependent cellular cytotoxicity (ADCC), antibody dependent cellular phagocytosis (ADCP), and antibody dependent complement deposition (ADCD) (43, 44).

As a long-term source of protective antibody, generation of robust LLPC responses is a primary goal of vaccination. However within the field, there is limited understanding of LLPC generation and differentiation from GC reactions as well as their migration, niche homing, and maintenance (6). The exit of ASC from secondary lymphoid organs is influenced by the expression of S1P1 (S1P receptor 1) as well as chemokine receptors CXCR3, CCR9, CCR10, and CXCR4. However, the regulation of expression of these chemokine receptors on ASC remains unclear (45). The underlying mechanisms and signals dictating LLPC precursor vs short-lived plasma cell (SLPC) generation within GCs and factors that can modulate this process remain largely unknown (46). Additionally, there is limited data to determine the duration of GC reactions needed to drive LLPC generation. There are limited studies on clonal dynamics linking LLPC clones back to earlier activation events happening within GCs (6), however, in one such study proliferation of PC precursors was found to be dependent on expression of TIGIT (T cell immunoreceptor with Ig and ITIM domains) (47). TIGIT is an inhibitory receptor expressed on subsets of T cells, natural killer cells, and B cells and is an emerging target for cancer immunotherapy (47–50). This same study also revealed PC precursors arising later in GC responses (i.e. day 35 post immunization) give rise to longer-lived BM PCs than PC precursors from early (i.e. day 21 post immunization) GC responses. These findings suggest that on-going GC responses driven by antigen availability can dictate PC precursor quantity and quality (47). Understanding GC mechanisms driving durable immunity is a focus that is particularly at the forefront of vaccinology given the recent deployment and widespread adoption of novel vaccine platforms such as LNP-mRNA vaccinations against SARS-CoV-2 that exhibited waning of neutralizing antibody leading to breakthrough or reinfections (17–19).

## Current understandings of factors impacting LLPC fate decision making

The signals that dictate differentiation of activated B cells into either memory B cells or LLPCs have been partially characterized but have yet to be fully elucidated (51). Factors that have been demonstrated to impact LLPC differentiation are strength of initial BCR-antigen interaction and BCR affinity (52), BCR signaling (53), cytokine signals, toll-like receptor (TLR) engagement (54), and CD40 ligation induced signaling (55).

The strength of the initial antigen-BCR interaction is dictated by both affinity and epitope density. Within secondary lymphoid organs, strong antigen binding induces B cells to become terminally differentiated extrafollicular plasmablasts, whereas weak antigen binding promotes entry into the GC for further affinity maturation (52). Through affinity maturation, B cells expressing BCRs with varying antigen affinities will emerge from GC reactions. B cells with lower antigen affinity in the GC are preferentially selected into the memory B cell compartment (56–59). In contrast, B cells with high antigen affinity in the GC are actively selected into the PC compartment (42). Though the notion of LLPCs arising from high affinity GC B cells clones is generally accepted, other studies have demonstrated affinity independent models of LLPC differentiation with pre-LLPCs and other GC B cell subsets exhibiting similar antigen affinity. To this end, pre-LLPCs were found to not preferentially acquire affinity-boosting mutations when compared to other GC B cell subsets (53, 58).

Differential BCR signaling also contributes to LLPC fate decision making (60). In GC B cells, BCR signaling is attenuated compared to naïve B cells, leading to minimal activation of transcription factor nuclear factor-KB (NF-KB) and altered phosphatidylinositol-3-OH-kinase (PI3K)-AKT signaling. Altered PI3K-AKT signaling leads to increased phosphorylation of the transcription factor FOXO1 known to play an essential role in antigen-driven GC B cell selection (60, 61). It has been proposed that attenuated BCR signaling in GC B cells favors positive selection but not differentiation enabling GC expansion while simultaneously preventing the premature development of long-lived progeny before affinity maturation has occurred (60). In line with this, the transcription factor BLIMP-1 is associated with commitment to PC fate (62, 63) and is responsible for the downregulation of several genes encoding BCR signaling components including CD19, CD45, syk, and BLNK (64, 65). In addition to its role in downregulating BCR signaling genes, BLIMP-1 is also involved in LLPC maintenance (66) and is thought to contribute to the metabolic restructuring of LLPCs, allowing for secretion of high amounts of antibody (67, 68). BLIMP-1, however, is not unique to LLPCs and is also expressed in plasmablasts. Within plasmablasts, BLIMP-1 has been shown to silence genes associated with antigen presentation and class switch recombination while promoting migration and adhesion (69).

Cytokines such as IL-2 (68), IL-10 (70), and IL-21 (71, 72) have been shown to promote differentiation of MBC or GC B cells into PCs. IL-2 has been shown to promote the expression of BLIMP-1 in GC B cells driving terminal differentiation into PCs (68). IL-2 as well as IL-10 has been demonstrated to enhance IL-21-mediated B cell proliferation, class switch recombination, and PC differentiation. IL-21 has been found to induce BLIMP-1 and activation-induced cytidine deaminase expression as well as enhance antibody production (71, 72). Similar to IL-2, IL-10 can induce MBC differentiation into PCs (73) and has been found to be directly produced by cells within the BM as well as gut PCs (74, 75). IL-10 as a regulatory cytokine could be contributing to an anti-inflammatory environment presumably allowing BM and gut IL-10 producing PCs to survive in these tissues for long periods of time (75). IL-10, as well as IL-2 and IL-21, have been shown to work in coordination with CD40 and BCR signaling to promote PC differentiation (54, 70).

TLR engagement has also been demonstrated to impact LLPC development. PCs have been shown to express all TLRs except for TLR10 and engagement of TLRs can influence antibody quantity and isotype. For example, TLR triggering leads to increased production and secretion of IgM and IgG by PCs. The production of IgM vs IgG by PCs has also been found to be dependent on which TLRs are engaged. Of note, TLR expression in B cells do differ between that of human and mice. Human B cells express limited to no TLR4 as opposed to mouse B cells. Human B cells in many cases replace TLR4 expression with TLR7 and TLR9 (76, 77). TLR1–4 engagement leads to preferential production of IgM while TLR7/8 triggering leads to IgG production in terminally differentiation PCs from peripheral blood (78). Studies have also demonstrated that antigens that engage both BCR and TLR7 promote differentiation into antigen-specific ASCs that express CD138, suggesting a LLPC phenotype (79). The importance of TLR signaling in LLPC development is exemplified by the small molecule TLR7/8 agonists R848 and 3M-052 having robust adjuvating activity. R848 and 3M-052 as an adjuvant for HIV targeting vaccines have been demonstrated to induce persistent antigen-specific BM LLPCs in non-human primates (NHPs) (80). R848 has been shown to mimic the impact of T cell dependent CD40 signaling by driving antibody secretion, cytokine production, protection from apoptosis, and upregulation of other costimulatory molecules such as CD80 (81).

Studies have demonstrated that strong CD40 stimulation induced by TFH cells is required for the development of memory B cells with high CD80 expression leading to preferential differentiation into PCs. NF-KB signaling and downstream of CD40 signaling was found to contribute to differential CD80 expression, with stronger CD40 signaling driving increased CD80 expression on B cells (82). CD40 ligation and downstream signaling, along with IL-21 and attenuated BCR signaling, has been shown to commit GC B cells toward LLPC fate (53, 60). Disruption of CD40 signaling particularly early after vaccination (1–3 days) inhibits GC formation and subsequent lowering of serum antibody responses. Later after vaccination (6–10 days) however, disruption of CD40 signaling has limited impact on serum antibody (83). CD40 signaling in turn has been described as being required but alone not sufficient to drive the formation of LLPCs (54).

In summary, factors that have been demonstrated to impact LLPC differentiation include strength of initial BCR-antigen interaction and BCR affinity (52), BCR signaling (53), cytokine, TLR engagement (54), and CD40 ligation induced signaling (55). These factors are likely functioning as part of a combinatorial signaling input specifically driving PC fate commitment, though the timing and interdependencies of these remain unclear (46). Genetic time-stamping methodologies as well as emerging techniques to study LLPCs that are outlined in more detail below, could be utilized to elucidate this. Although some aspects of signaling needed to drive LLPC differentiation have been elucidated, there is likely a level of complexity within these varying signaling cascades that is under studied and therefore creates a gap in knowledge of how to design vaccines in a way to induce LLCP fate decision making and durable immunity (23).

## Challenges of studying LLPCs

As opposed to other B cell subsets, LLPCs pose unique technical and biological challenges which makes the study of this cell type particularly difficult. Even in the BM where they are most abundant, LLPCs make up less than 1% of total cells in the BM with antigen-specific LLPCs for a given antigen of interest making up an even lower frequency (23, 25). Consequently, collection of sufficient LLPC cell numbers to meet the limit of sensitivity of immunological assays is a challenge. However, this can in part be mitigated through enrichment and pooling strategies. Magnetic bead-based cell isolation can be used to either positively or negatively select B cells or PCs using a combination of biotinylated antibodies and streptavidin coated magnetic beads. Selection kits are commercially available for mice as well as humans to isolate B cells expressing Sca-1 and CD138 (84, 85). Pooling of BM from several individual mice is a feasible means to increase LLPC cell numbers. However, sample pooling can mask biological variability (85).

CD138, or syndecan-1, is a cell surface proteoglycan containing heparan sulfate chains that mediate cell-to-cell adhesion. These heparan sulfate chains bind to cytokines like IL-10, which has been shown to promote PC differentiation (86, 87). The increased expression of CD138 on PCs may serve to regulate the availability of cytokines such as IL-10 needed for differentiation (73). In addition to CD138, Sca-1 expression is also upregulated on PCs. Sca-1, also known as Ly-6 A/E, is a GPI-linked cell surface protein that is known to play a role in hematopoietic stem cell lineage choice (88). Animals deficient in Sca-1 exhibit decreased megakaryocyte and platelet formation (89). Both megakaryocytes and platelets have been recently linked to enhanced PC survival, antibody production, and vaccine induce antibody durability. Enhanced survival and antibody production from bone marrow PCs was demonstrated in co-cultures with megakaryocytes in a contact dependent manner (90). These findings suggest that increased expression of Sca-1 on PCs mediates their survival through its interaction with megakaryocytes.

Although LLPCs upregulate CD138 and Sca-1 on the cell surface, these markers are not exclusively expressed on LLPCs (25). Other cell surface markers in addition to CD138 and Sca-1 have been characterized for LLPCs including CD38 and CD27, however, these can be expressed on other ASCs such as SLPCs and plasmablasts (3, 91–93). The ability to distinguish plasmablasts from short-lived and long-lived PC precursors remains a challenge (46). The lack of a clear distinctive phenotype makes it challenging to specifically isolate and study LLPCs. To this end, LLPCs are often operationally defined or implied by the longevity of the antibody response and presence within survival niches (92, 94, 95).

Sampling of survival niches where LLPCs reside is another challenge to studying this cell type. Longitudinal BM sampling in mice, although technically feasible, has low yields compared to terminal BM harvesting (96) thus limiting the ability to understand LLPC dynamics over time in mice without using a large number of mice in a given study. Longitudinal BM sampling therefore requires NHP or human subjects, both of which also present challenges. BM aspiration is an invasive procedure that can have associated complications, although rare, such as trauma to neighboring structures and tissues, infection, and hemorrhage (97).

Mouse models have been critical in advancing the current understanding of LLPCs. Recently the use of sophisticated genetically engineered mouse models has allowed for the *in vivo* labelling of PCs that can be traced over time, a method referred to as genetic time-stamping (95, 98, 99). Using an inducible Cre-Lox system to drive expression of fluorescently labeled BLIMP, a key transcription factor in PC fate commitment (62, 63), has allowed for deeper understanding of LLPC turnover, tissue origin, and trafficking in both homeostatic conditions (95, 98) as well post-vaccination (99, 100). These studies have revealed homeostatic turnover of LLPC as being intrinsically determined (95), with longevity being influenced by factors such as immunoglobulin isotype (98), rather than being dictated by niche competition. Additionally, PC within the BM were characterized as a compendium of PC from varying tissues of origin (i.e spleen, lymph nodes, colon) with tissue origin being reflected in their transcriptional profile (98). Using genetic time-stamping in combination with vaccination revealed that the LLPC pool (B220^lo^MHC-II^lo^) was seeded from both GC-independent as well as GC-dependent PCs. Additionally, antigen-specific B220^lo^MHC-II^lo^ cells were found to better mobilize to the BM niche and exhibit better survival potential compared to antigen specific B220^Hi^MHC-II^Hi^ (99). The learnings from studies using both genetic time-stamping and immunization were performed using either hapten antigen or model antigens (99, 100). Although useful, these antigens are limited in size and complexity and therefore may not be fully reflective of LLPC responses driven by complex antigens such as trimeric fusion proteins. Novel genetic time-stamping mouse models have led to important findings, however, there remain limitations for studying LLPCs in mice. LLPCs in mice have been demonstrated to survive for over a year, however, the total life span of mice is 1–3 years. The drastically shorter life span of mice compared to humans makes the study of immunological persistence of vaccine responses and LLPCs challenging (25, 63).

ASCs isolated from BM of mice and humans have been demonstrated to undergo rapid apoptosis ex vivo making the *in vitro* study of ASCs such as LLPCs difficult (24, 101–103). Studies utilizing metabolomics, transcriptomics, and proteomics have identified critical survival factors to recapitulate the BM niche *in vitro* (30, 45). An *in vitro* system using a combination of soluble secreted factors from primary BM mesenchymal stromal cells, exogenous addition of a proliferation-inducing ligand (APRIL), and hypoxic conditions has demonstrated survival of ASCs in culture for up to 56 days (24, 30). This system has been used to evaluate the morphology, transcriptomes, and epigenetics or human blood ASCs compared to BM LLPCs (24). The BM consists of many different cell types such as stromal cells, eosinophils (104), megakaryocytes (105), basophils (106), monocytes (107) and dendritic cells (108) all of which can provide surface ligands as well as secreted signals to promote LLPC survival (30). Given recent findings that megakaryocytes enhance bone marrow PC survival (90), the addition of megakaryocytes into *in vitro* systems that mimic the BM niche may be able to provide prolonged survival as well better represent additional cell types within the BM. Although a useful tool for investigating differentiation and maturation programs of early ASCs to LLPCs, this *in vitro* system is likely not fully capturing the complexity of the BM niche. Building upon this *in vitro* system and combining it with the novel technologies and techniques outlined below (i.e. Nanovials or TRAPnSeq) could help in elucidating critical surface ligands and additional secreted signals by other cell types within the BM niche that would be needed for LLPC survival. Vaccines formulated with ligands or signaling molecules identified using these systems have the potential to strategically drive LLPC survival and therefore durable immunity.

## Emerging novel technologies to study plasma cells

Despite the technical and biological challenges LLPCs study poses, substantial efforts have been made to develop novel technologies and techniques to better understand these cells. Due to the high sensitivity and reliability of B cell enzyme-linked immunosorbent spot assays (ELISpot), this assay has traditionally been used for the quantification of antigen-specific plasma cells (109, 110). However, a major limitation to ELISpot assays is the lack of downstream characterization of cells and secreted antibodies (29).

**Figure 1:**
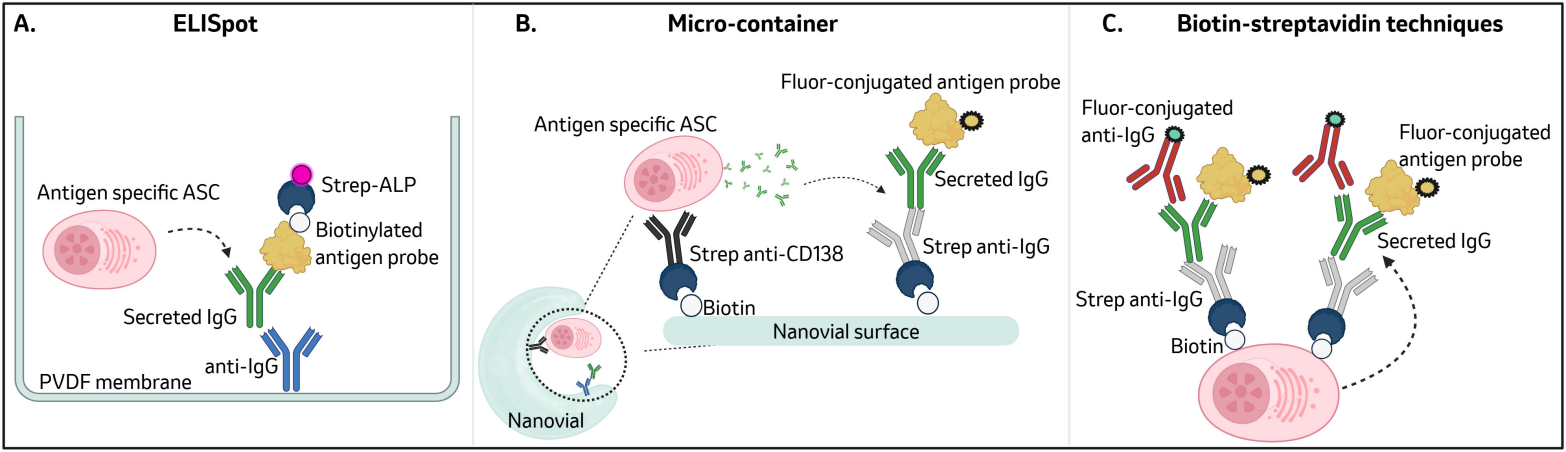
Methods to detect antigen-specific LLPCs: ELISpot assays are a highly sensitive method for detection of antigen-specific LLPCs but lack the ability for downstream characterization (29, 109, 110) **(A)**. Micro-containers (i.e. Nanovials) use functionalized hydrogels to capture a single LLPCs and its secreted antibody. Nanovials are compatible with commercial fluorescence-activated cell sorters and single-cell transcriptomic systems such as the 10x chromium system (26, 117) **(B)**. Biotin-streptavidin techniques (i.e. Ig Trap, TRAPnSeq) capture secreted antibody from LLPCs by biotinylating the cell surface and tethering a streptavidin-conjugated anti-Fc IgG. These techniques are also compatible with downstream FACS and single cell sequencing (28, 29) **(C)**. Figure was created in Biorender.

Multi-color flow cytometry, fluorescence-activated cell sorting (FACS), and single-cell RNA sequencing technologies have been utilized to gain a deeper understanding of antigen-specific MBC (32, 111). MBC are well suited for these technologies as they express traditional B cell lineage markers (i.e. CD19), memory markers (i.e. CD38, CD27) and antigen-specific BCRs on their surface (112). In contrast, LLPCs express little to no B cell lineage markers or BCRs on their surface (23). Surface expression of CD38 (3), CXCR4 (113), CD27 (114), Sca-1 (115), and CD138 have been used to identify PCs in mice and humans. Recently, surface CD102 expression has been identified as a biomarker for LLPCs in mice, NHP, and humans (23). These surface markers, although helpful for identifying LLPCs, do not inform on the antigen specificity of the antibody being secreted by LLPCs. To this end, several novel emerging technologies and techniques summarized in **Figure 1** have been utilized to link LLPC surface marker expression with secreted antibody on a single cell level.

**Figure 2:**
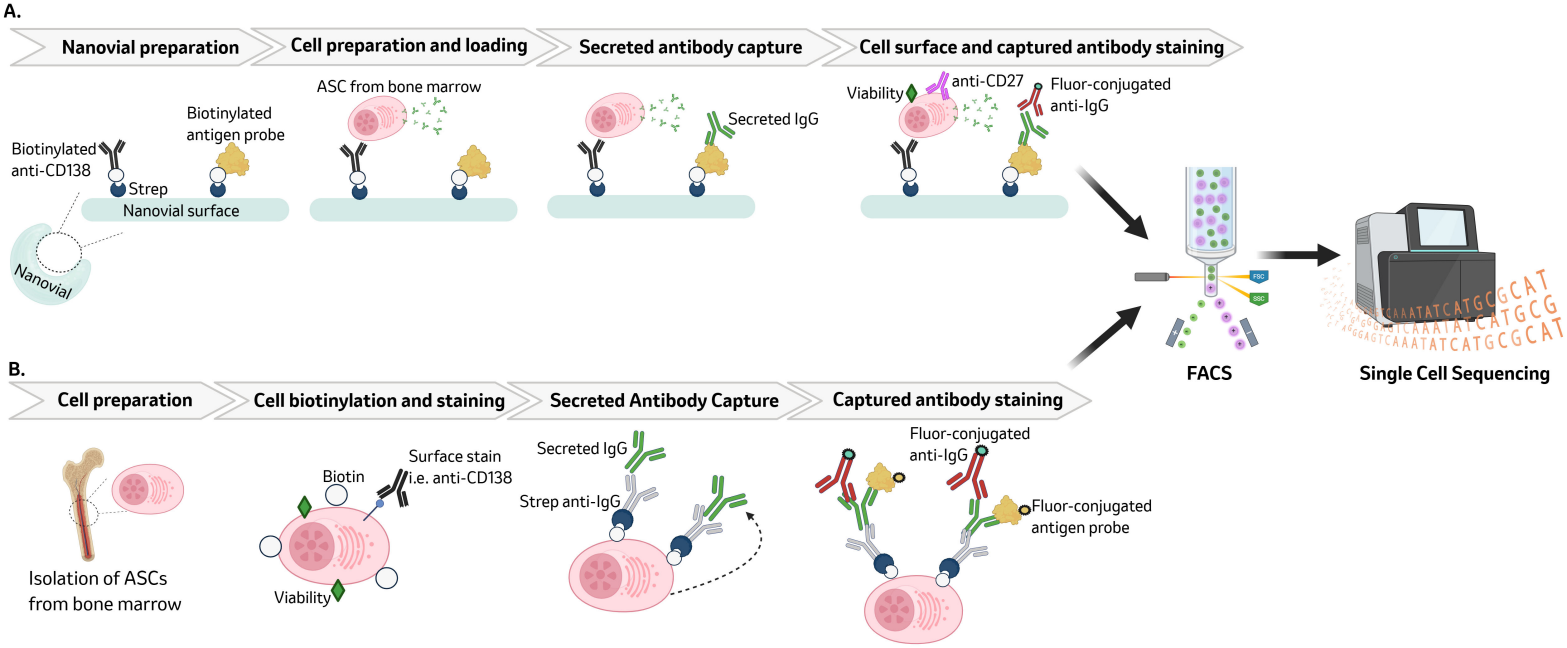
Workflow overview for Nanovial micro-container and biotin-streptavidin techniques: Nanovials are prepared by incubating streptavidin, biotinylated anti-CD138, and biotinylated antigen. LLPCs are loaded into the nanovials and incubated to allow for secreted antibody capture. Following antibody capture, LLPCs are surface stained and capture antibody is stained using a fluor-conjugated anti-IgG (117) **(A)**. Biotin-streptavidin techniques such as Ig trap and TRAPnSeq biotinylate the surface of LLPCs. Cells are then surface stained before addition of streptavidin-conjugated anti-IgG to capture secreted IgG. Secreted IgG tethered to the LLPC can then stained using an fluor-conjugated anti-IgG and fluor-conjugated antigen (28, 29) **(B)**. Both methods allow for downstream characterization of antigen-specific LLPCs via FACS and single cell sequencing. Figure was created in Biorender.

One such technology is Nanovials, which are hydrogel-based chemically functionalized microcontainers that serve to capture a single cell and its secreted proteins. The bowl-shaped cavity in nanovials is coated with either streptavidin or biotin and functionalized with biotinylated or streptavidin-conjugated antibodies targeting surface proteins (i.e. CD38, CD138, CD27), antigen, or anti-IgG. This will enable the capture LLPCs and their secreted antigen-specific IgG within the Nanovials (26, 29). Individual cells are loaded into Nanovials by following Poisson statistics and using a ratio of cells to Nanovials that leads to 10–20% of Nanovials being single cell-loaded. Solutions containing fluorescently conjugated or oligo-barcoded antibodies can be exchanged within the Nanovials enabling additional phenotypic as well as functional (i.e. antibody secretion) characterization of nanovial-captured cells (26). To this end, single LLPCs and their secreted antibodies that have been captured within the Nanovials can then be stained using antibodies against surface markers or antigen probes that are fluor-conjugated or oligo-barcoded. Nanovials are compatible with commercial flow cytometers, sorters, and single cell sequencing instruments. A technique referred to as secretion with single-cell sequencing (SEC-seq) has combined Nanovials, FACS, and single cell sequencing to genomically profile antigen specific LLPCs (26, 116, 117). A graphical summary of a SEC-seq workflow is shown in **Figure 2A**.

A similar technology to the Nanovials, called Nanorods, has also been recently developed. Nanorods, though more poised for antibody discovery and less so LLPC characterization, are like Nanovials in that they encapsulate a single ASC within a microgel and capture its secreted antibodies (27). In contrast to Nanovials, the microgels are not pre-coated thus do not allow for the selective capturing of LLPCs as opposed to other ASCs (26, 27). The microgels can be functionalized with antigen-coated magnetic Nanorods allowing for the magnetic separation and selection of microgels containing a cell making antibody of a desired antigen specificity (27). The ease of magnetic separation as opposed to FACS is a desirable quality of the Nanorods though further of development of these microgels to specifically capture LLPCs would be needed to utilize this platform for LLPC characterization.

Outside of novel technologies, creative techniques have also been recently described to identify and genomically characterize antigen-specific LLPCs using existing FACS and single cell sequencing technologies. A workflow summary of these biotin-streptavidin based techniques is represented in **Figure 2B**. Such techniques, referred to as Ig Trap (29) or TRAPnSeq (28), work by using biotin-streptavidin interactions to trap the secreted immunoglobulins (Igs) onto the surface of the LLPC it is being secreted from. This is achieved through biotinylation of cell surface proteins on bone marrow mononuclear cells (BMMCs). Biotinylated cells are viability stained and for additional surface markers prior to addition of a streptavidin conjugated anti-Ig Fc antibody, which will be tethered to the biotinylated cell surface. In this manner, a pseudo-BCR is created and used to capture Igs secreted by the LLPC by binding to the Fc region of the secreted Igs. This allows for the antigen binding domains of secreted and captured Igs to remain accessible. Subsequent incubation with a fluorescently tagged anti-Ig specific for heavy and light chains as well as antigen probe allow for identification of LLPCs that are secreting Igs specific to an antigen of interest. These methods have been used to identify antigen specificity of IgG (29) and IgE (28) secreting LLPCs. Currently these methods have been demonstrated using BMMCs from NHP (29) and mice (28) but have yet to be applied to human BMMCs. There are limited laboratory groups that have had success with these methods (28, 29) suggesting a level of nuance and complexity to these assays.

Collectively, these novel technologies (Nanovials and Nanorods) and techniques (SEC-Seq, Ig Trap, and TRAPnSeq), show promise in their ability to enable genomic profiling of LLPCs at a single cell level and in an antigen-specific manner. The use of these novel technologies and techniques with human clinical samples or with genetic time-stamping mouse models would be an extremely powerful tool to build on current knowledge as well as overcome some of the technical and biological challenges of LLPCs. For example, the sequencing of PCs in genetic time-stamping models have all been performed on a bulk level (95, 98), therefore masking any clonality differences that could be elucidated by single cell sequencing. Bulk sequencing, although helpful for making general conclusions, is not ideal for LLPCs given the heterogeneity of these cells and lack of distinctive phenotype (94). Techniques such as Ig Trap combined with genetic time-stamping would not only capture the heterogeneity of LLPCs but could genomically characterize these on a single cell level which could lead to identifying a clearer, more unique phenotype for LLPCs. These novel technologies and techniques can further elucidate isotype-specific PC heterogeneity (94), which in the context of vaccination, would be particularly powerful in understanding how to drive IgA LLPCs to protect against mucosal pathogens. This level of LLPC profiling can also be used to build on the current understanding of key factors driving LLPC fate decision making (i.e. BCR signaling (53), TLR engagement (54)) and enable identification of novel molecular pathways dictating LLPC fate decision making. Once identified, these molecular pathways orchestrating LLPC differentiation can be targeted in vaccine design to strategically induce durable immunity.

## Impact of vaccine design decisions on humoral and B cell immunological memory

During vaccine development, there are several design decisions that need to be made that impact vaccine induced humoral and B cell immunological memory. These decisions include but are not limited to vaccine platform (7), adjuvant (8), antigen valency (9), duration of antigen bioavailability (10), and route of administration (11). In this section we will review how each of these vaccine design decisions have been demonstrated to impact immunological memory with a focus on humoral and B cell responses.

### Vaccine platform

**Figure 3:**
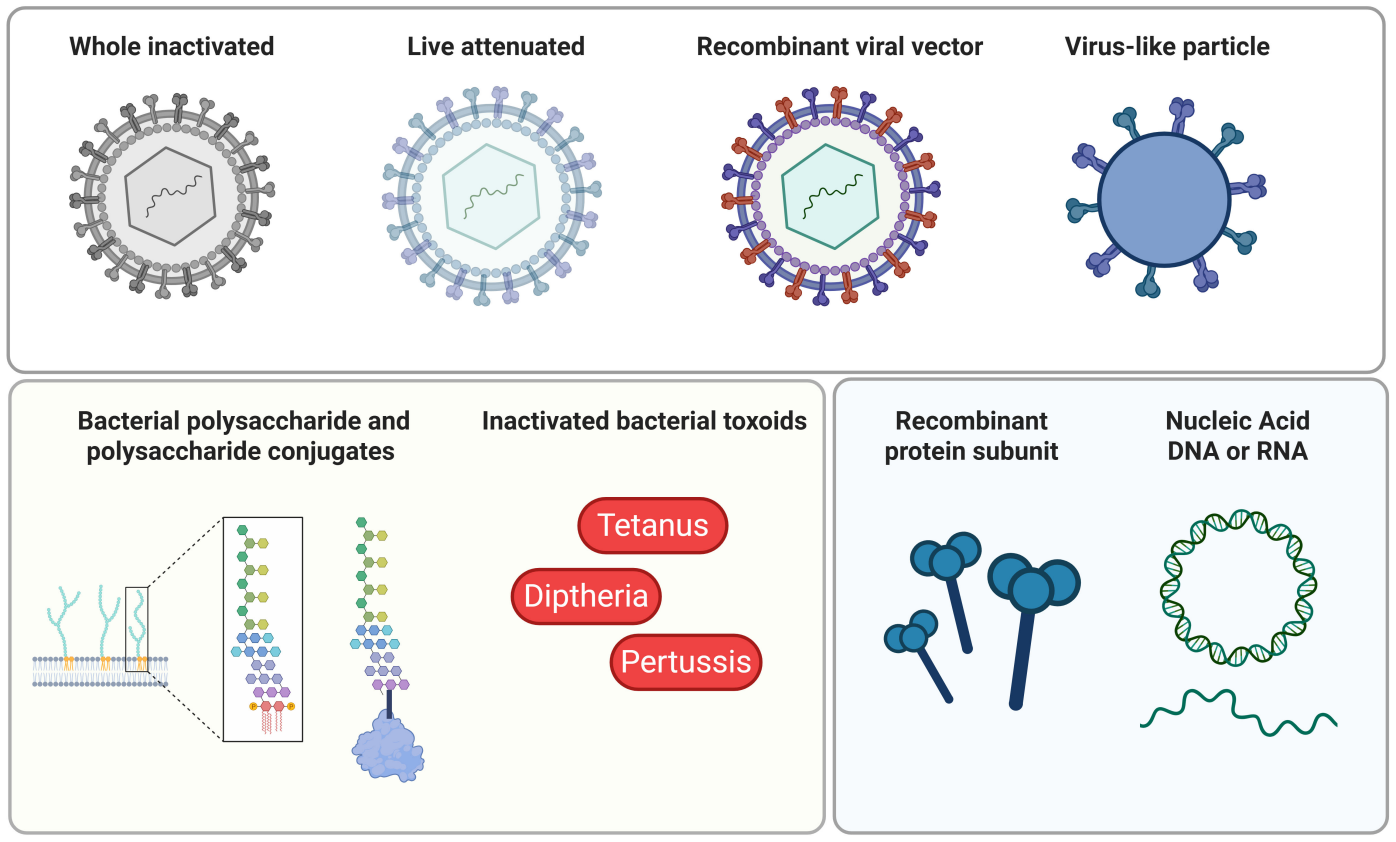
Summary of clinically approved vaccine platforms: Vaccine platforms approved for clinical use range from whole organisms that have been inactivated or attenuated, vector systems, such as recombinant viral vectors and virus-like particles (VLPs), bacterial polysaccharides and polysaccharides conjugate vaccines, inactivated bacterial toxoids, recombinant protein subunit, and nucleic acid (DNA or RNA). All of these can be used to induce antigen-specific immune responses (118, 119). Figure was created in Biorender.

Vaccine platforms currently approved for clinical use include whole inactivated, live attenuated, recombinant viral vectors, virus-like particles, recombinant protein subunit, nucleic acid (DNA and mRNA), inactivated bacterial toxoids, bacterial polysaccharides and polysaccharides conjugate vaccines (see **Figure 3**) (118, 119). Of these platforms, live attenuated vaccines developed to protect against yellow fever, smallpox, as well as measles, mumps, and rubella, have been demonstrated to induce lifelong immunity (4, 17, 90, 120). Similarly, virus-like particle vaccines against human papillomavirus, such as Gardasil^®^9 and Cervarix, have also demonstrated stable and durable protective antibody titers that last for more than 10 years post-vaccination (13–16).

In contrast, bacterial toxoid vaccines such as diphtheria, tetanus, and pertussis (DTaP and Tdap) vaccines have demonstrated waning immunity, resulting in increased disease incidents rates and requiring repeated booster immunization (4, 20, 21). Similarly, waning immunity has also been observed with the recent deployment of mRNA vaccines against SARS-CoV-2, which exhibit a decrease in neutralizing antibody 3–6 months post vaccination leading to breakthrough or reinfection (17–19, 121).

In trying to understand why live attenuated vaccines induce longer lived immune responses compared to toxoid or mRNA platform vaccines, it is important to consider that live attenuated vaccines, in contrast to toxoid or mRNA vaccines, contain the whole pathogen allowing for pathogen-associated molecular patterns (PAMPs) to trigger and activate several TLRs simultaneously, suggesting this as a possible means for achieving durable humoral immunity (122, 123). PAMP recognition driven by live attenuated vaccines can induce innate cell trained immunity and therefore innate cell immunological memory (124). TLR signaling, particularly TLR7/8, has been shown to induce persistent antigen-specific BM LLPCs, demonstrating the importance of TLR7/8 signaling in LLPC development (80). An additional key difference of live attenuated vaccines compared to toxoid or mRNA vaccines is the ability of live attenuated vaccines to replicate in the host. Low level replication by live attenuated vaccines likely induces additional TLR signaling as well as engagement other pattern recognition receptors (PRRs), such as cytosolic sensors RIG-1 and Mda5 (122, 125). The wide range of immune responses driven by live attenuated vaccines are likely contributing to the associated humoral durability with this platform as opposed to others. It is clear that different vaccine platforms induce varying degrees of immunological memory however the molecular mechanisms underpinning this are not fully understood.

### Vaccine adjuvants

Depending on the vaccine platform used, adjuvants may be required to generate sufficient immunogenicity for protective immunity. Currently there are five main classes of adjuvants that are approved for clinical use including aluminum, oil-in-water emulsions, liposome-based, nanoparticles, and pattern recognition receptor (PRR) engagers (126). A brief summary of these adjuvants and their impact on humoral and B cell immunological memory will be described here. Adjuvant mechanism as it relates to B cell responses will also be discussed here. Summaries of adjuvant mechanism as it relates to other immune cell subtypes can be found in depth in other reviews (126–128).

Aluminum is the most widely and longest used vaccine adjuvant with its original licensure in the 1930s. Aluminum as an adjuvant is known to enhance humoral responses through its ability activate the inflammasome and slowly release vaccine antigen from the site of immunization (126, 129–131). Studies using site-specific modified antigens with short peptides composed of repeating phosphoserine demonstrate enhanced binding to aluminum. This enhanced aluminum binding, and therefore increased antigen bioavailability, elicited increased antibody magnitude and quality (neutralization), GC and memory B cells, and LLPC responses compared to conventional antigen-aluminum adsorption (131, 132). These studies demonstrate that the antigen depot effect mediated by aluminum adjuvants can drive humoral and B cell immunological memory.

MF59 and AS03 are oil-in-water emulsion adjuvants with slightly different compositions but drive similar immune responses. Both MF59 and AS03 are squalene emulsions but AS03 contains alpha-tocopherol as an additional immunostimulant component (128). Oil-in-water emulsion adjuvants have been demonstrated to enhance antibody magnitude, quality, isotype switching, and breadth (133), as well as cytokine production such as IFN-γ and IL-2 (134). IL-2 plays a role in B cell responses by promoting the expression of BLIMP-1 in GC B cells driving terminal differentiation into PCs (68). Additionally, oil-in-water emulsion adjuvants induce antigen-specific GC B cells that can persist for several months post immunization (133). Enhancement of humoral and B cell immunity by oil-in-water emulsions is likely from the slow release of antigen into lymph nodes resulting in prolonged antigen bioavailability and increased antigen presentation (128, 135).

Liposome based adjuvants AS01 and AS04 both contain monophosphoryl lipid A (MPLA), a detoxified form of lipopolysaccharide (LPS) but differ in that AS01 also contains Quillaja saponaria molina (QS-21), a saponin extracted from the *Quillaja soponaria* tree, while AS04 contains alum (136–138). MPLA in both AS01 and AS04 activates TLR4. TLR4 signaling has been shown to enhance B cell trafficking to lymph nodes, sustained B cell proliferation, and enhance the generation of MBC and PC (139). The ability of liposome-based adjuvants to enhance antibody and MBC is likely due to rapid activation and trafficking of APCs to lymph nodes that drive subsequent T and B cell activation (140, 141).

Nanoparticle adjuvants, such as Matrix M and lipid nanoparticles (LNPs), were recently approved adjuvants for use in SARS-CoV-2 and malaria targeting vaccines. LNPs can serve as delivery systems for its mRNA cargo while also having adjuvating properties mediated by ionizable lipids. Studies using empty LNPs formulated with recombinant hemagglutinin protein identified enhanced antibody magnitude, isotype switching, and B cell subsets (GC, memory, and PCs) compared the hemagglutinin protein alone demonstrating the adjuvating ability of the LNP itself (126, 142). Additional studies demonstrate empty LNPs can drive DC maturation and activation leading to production of cytokines such as IL-21 and CD40L (143). Although IL-21 (71) and CD40 (60) signaling has been shown to impact LLPC development, currently licensed SARS-CoV-2 and malaria targeting vaccines adjuvanted with Matrix M or LNPs require booster doses to maintain efficacy (121, 144), suggesting that LLPCs may not be developed following immunization.

Pattern recognition receptor (PRR) engaging adjuvants include virosomes, outer membrane vesicles (OMV), and CpG. Virosomes are enveloped virus-like particles containing viral proteins that act as both an adjuvant as well as an antigen delivery vector. The envelope of virosomes is derived from native influenza virus envelope (i.e. envelope glycoprotein and haemagglutinin) (127, 147). This property enhances antigen-containing virosome uptake by APCs (146). Similar to virosomes, OMVs can serve as an antigen delivery vector with adjuvanting properties. OMVs are derived from the outer membrane of gram-negative bacteria and contain several bacterial antigens and proteins that drive TLR signaling (126, 147). CpG is made up of cytosine phosphoguanine (CpG) motifs which are synthetic oligodeoxynucleotides that mimic bacterial DNA to drive TLR9 signaling (126, 148). In humans, TLR9 expression is restricted to plasmacytoid DCs and B cells (149), suggesting the enhanced B cell activation, proliferation, antibody secretion, and protection from apoptosis from CpG (150) can be mediated by TLR9 signaling in plasmacytoid DCs or B cells.

The choice of adjuvant in vaccine design can be used to increase the magnitude and durability memory immune cell formation (127). Studies evaluating varying adjuvants with the same protein subunit vaccine platform demonstrate a differential impact of adjuvant on antibody magnitude, antibody quality, and memory B cell formation (8, 151). Regarding adjuvant choice on LLPC development, though not clinically approved, the TLR7/8 agonist adjuvant 3M-052 is a promising candidate for driving durable antibody responses as it has been shown to induce persistent LLPCs in NHPs (80).

### Antigen valency and bioavailability

Increasing antigen valency and duration of antigen bioavailability is based on principles of mimicking natural infection which can be incorporated into vaccine design to impact immunological memory formation (9, 10). Antigens with higher valency can be present in multiple copies are capable of binding and crosslinking multiple BCRs, resulting in enhanced BCR avidity and sustained BCR signaling (152). This notion applies to both viral and bacterial pathogens which exhibit surface antigens that are highly repetitive and highly structurally organized (12). Antigen valency has been strongly associated with higher antibody titers in the context of both viral infections as well as vaccine immune responses (9). Studies using both small chemical haptens (153) or peptides (154), as well as complex trimeric proteins such as human immunodeficiency virus (HIV) envelope (9, 155), demonstrate the impact of high antigen valency on germinal center formation, memory B cell generation, serum antibody, and LLPC responses. The importance of antigen valency on immune durability can be further exemplified by the multivalent nature of HPV-VLP vaccines and their demonstrated ability to induce stable and durable protective antibody that last for more than 10 years post vaccination (13–16).

During acute natural infections, the immune system is exposed to escalating antigen and inflammation over the course of days to weeks. This leads to sustained antigen bioavailability that can participate in GC reactions and drive B cell affinity maturation (10, 156). Studies have shown that exponentially increasing vaccine dosing kinetics leads to enhanced capture and retention of vaccine antigen within lymph nodes driving increased GC B cell expansion and PC generation compared to a single bolus vaccine dose (10, 157). Duration of antigen bioavailability and its impact on antibody quality is further exemplified in the development of broadly neutralizing antibodies (bNAbs) against HIV (158). BNAbs require a high level of affinity maturation that is in part driven by chronic antigen exposure (159). Though duration of antigen bioavailability has clearly demonstrated impacts on GC reactions and antibody quality, the impact of antigen bioavailability on LLPC development specifically has yet to be elucidated.

### Route of administration

Vaccine route of administration can impact immunological memory and can be strategically exploited to induce immunity at sites of pathogen entry and infection. Most vaccines are administered via intramuscular (IM), subcutaneous (SC), or intradermal (ID) injection. Studies have shown that SC immunization can enhance the rate of development and magnitude neutralizing antibodies compared to IM immunization (11). Though commonly used, IM, SC, and ID injection induce limited mucosal immunity. Since many pathogens enter the human host through mucosal membranes in the respiratory, digestive, and genital tracts, vaccines that can induce immunity at mucosal sites are likely to enhance protection against infection and reduce transmission (160). In line with this, vaccine administration routes targeting mucosal sites such as intranasal (IN), pulmonary delivery via aerosol inhalation (161, 162) or oral administration have been explored to enhance mucosal immunity. Strategies using an IM prime followed by an IN boost have demonstrated robust induction of systemic and mucosal neutralizing antibodies that were durable and protective (163). Human studies evaluating route of administration on influenza specific IgA and IgG secreting cells found intranasal immunization induced mainly IgG secreting B cells while oral administration induced IgA secreting B cells (164, 165). However, these ASCs were from peripheral blood and antibody levels were not measured longitudinally suggesting these ASCs are not part of the LLPC compartment. Though these findings suggest the modulation of antibody isotype based on immunization route (165, 166), the impact on administration on LLPC development has yet to be characterized.

### Vaccine design decisions to drive LLPC responses

Of the vaccine design decisions outlined above, it remains unclear which of these or combination of these are most critical for the development of antigen-specific LLPC responses. Combining these novel technologies (i.e. Nanovials and Nanorods) and techniques (i.e. SEC-Seq, Ig Trap, and TRAPnSeq) with varying aspects of vaccine design (i.e. varying antigen valency, adjuvant, or vaccine platform) can characterize antigen-specific LLPC responses, the clonality of those responses, and therefore may differentiate which vaccine design aspects induces the most robust LLPC response. This approach could also elucidate if varying vaccine design aspects induce differential isotype-specific LLPCs. Vaccines could therefore be strategically designed to induce, for example, IgA LLPCs or IgE LLPCs to mediate durable protection against mucosal targeting pathogens (167) or helminths (168) respectively. Similarly, these novel technologies and techniques can be combined with vaccines of varying antigen valency, varying adjuvant, or similar antigen but varying platform to understand how these vaccine design decisions influence the development of antigen-specific LLPC responses.

## Heterogeneity of LLPCs in vaccine responses

Studies in both mice (47, 94, 169) and humans (3, 22) have revealed vaccination can induce heterogenous populations of PCs and LLPCs. The heterogeneity of PCs is thought to in part be dictated by immunoglobulin isotype, tissue of residence, and ancestral GC experience (45, 169). IgA-expressing LLPCs have been found to exhibit distinct transcriptomes from IgG and IgM LLPCs. This could be due to factors that drive isotype switching and transcriptome imprinting during PC differentiation or differential signaling provided by IgG and IgA receptors compared to IgM (94). LLPCs residing in the GALT predominantly express IgA, although IgA-expressing LLPCs can also be found within the BM (63, 170–172). The higher distribution of IgA expressing LLPCs within the gut and the transcriptionally distinct IgA LLPCs compared to other isotypes suggests unique differentiation of IgA LLPCs at the mucosa (94). In mouse models, IgA expressing PCs were found to be longer lived than IgM and IgG PCs with IgG PCs exhibiting the highest turnover and longevity differences being independent of tissue (98). In humans, IgA expressing PCs have been demonstrated to be maintained within the intestine for life, despite gut PC being previously considered as shorter lived than PCs within the BM (173). IgG and IgM LLPCs are both found within the BM, which could point to their shared similar transcriptomes, however IgG and IgM LLPCs differ in developmental origins with IgG-expressing LLPCs requiring GCs for their development while IgM-expressing LLPCs can arise in the absence of GCs (94, 174). There is increasing evidence that IgE expressing LLPCs can accumulate within the bone marrow of chronic allergen exposed mice (175) as well as in atopic humans (176). The finding of continued IgE production in the absence of allergen re-exposure (176–178) and limited success of therapeutic approaches to reduce serum IgE by targeting IgE class switching or IgE class switched cells (176, 179–182) suggests persistent IgE is stemming from IgE LLPCs. Limited studies have characterized the developmental pathway and transcriptional signatures of IgE PCs (175, 176) leaving much to be learned about the origin and maintenance of IgE LLPCs.

The heterogenous PC populations within the BM arise from numerous B cell progenitors through various activation routes and stages of differentiation. Mature B cells can be classified into two main lineages, B1 and B2 cells. B1 cells are innate-like B cells that are predominantly found in the peritoneal and pleural cavities and produce polyreactive, modest affinity antibody without prior antigen exposure therefore acting as immediate nonspecific defense against common bacterial pathogens (94, 183, 184). In contrast, B2 cells form follicles of secondary lymphoid organs and are responsible for generating specific antibody responses against foreign antigens, typically involving T cell–dependent BCR affinity maturation and somatic hypermutation (183, 185). Though PCs can arise from both B1 and B2 cells, it is thought that LLPC predominately arise from B2 cells supporting the notion of GC dependency for LLPC development particularly in the case of IgG LLPCs (169).

Bone marrow PCs and LLPCs arising from vaccination in humans have contributed to the understanding of vaccine-induced PC heterogeneity and longevity. One such study evaluated human subjects that had received tetanus toxoid vaccination, recent influenza vaccination, or childhood measles or mumps infection without vaccination. Tetanus-specific PCs were identified as CD19^-^CD38^+^CD138^+^ which also coincided with measles and mumps specific PCs CD19^-^CD38^+^CD138^+^ demonstrating the longevity of this subset (3). Despite shared PC expression of CD19^-^CD38^+^CD138^+^ in tetanus-specific and measles-specific PCs (3), the antibody decay rates against these pathogens drastically differ with models projecting measles antibodies last over 3000 years and tetanus antibodies last only 11 years (4). This could be due to measles infection or live viral vaccination inducing stronger innate signals, such as cytokines, which influence LLPC survival compared to tetanus toxoid vaccination. Though the role of individual cytokine promotion on BM PC survival is not fully understood (170), IL-21 initiates STAT-3 signaling (186) which influences the ability of PCs to respond to APRIL, a known component to PC survival (187). In contrast to CD19^-^CD38^+^CD138^+^ tetanus-specific and measles specific PCs, influenza-specific PCs were found across multiple PC subsets (CD19+ CD38^+^CD138+, CD19+ CD38+CD138-, and CD19^-^CD38^+^CD138^+^). These findings suggest that SLPCs may be found within multiple compartments (CD19^+^CD38^+^CD138^+^ and CD19^-^CD38^+^CD138^+^) while LLPCs are more restricted to CD19-CD38^+^CD138^+^ (3, 6, 45). In-line with these findings of SLPCs following influenza vaccination, a separate study demonstrated the disappearance of influenza-specific bone marrow PCs 1 year post influenza vaccination in human subjects. The clones that were lost over time were shown to be influenza vaccine-induced, rather than pre-existing, which substantiates the observation that influenza vaccine-mediated humoral immunity is not well maintained (6, 22).

## Conclusions

Vaccine-mediated protection relies on the generation of immunological memory and high affinity antibodies that can rapidly neutralize pathogens and mediate clearance (5, 23). LLPCs that are generated via natural infection or vaccine-induced GC reactions are known to be a source of long-term antibody that can be high affinity and neutralizing (3). Certain aspects such as strength of initial BCR-antigen interaction (52), BCR affinity and signaling (53), as well as cytokine, TLR engagement (54), and CD40 ligation induced signaling (55) that drive LLPC differentiation have been described. However, the mechanisms for generation of LLPC precursors within GCs and subsequent migration to survival niches remains largely unknown (6).

Substantial efforts have been made to elucidate key vaccine design decisions that impact broader immunological memory formation. These include vaccine platform choice (7), adjuvant selection (8), antigen valency (9), duration of antigen bioavailability (10), and route of administration (11). However, many of these efforts have focused on antibody and memory B cell development with limited study on how these vaccine design choices impact LLPCs specifically. The limited study of LLPCs is fueled by many challenging aspects of this cell type. LLPCs are extremely rare and located in survival niches that are not easy to sample (3, 22, 23). Additionally, LLPCs undergo rapid ex vivo apoptosis making *in vitro* study of these cells difficult (24). Heterogeneity within the PC compartment also makes defining a distinctive phenotype of LLPCs challenging (25).

There are several aspects of LLPCs that are not fully understood that can hinder the ability to strategically target their development in vaccine design. For example, there is limited understanding of LLPC generation and differentiation from GC reactions as well as their migration, niche homing, and maintenance (6). Migration, in part, has been described through expression of chemokine receptors (i.e. CXCR3, CCR9, CCR10, and CXCR4) however the regulated expression of these remains unclear (45). Additionally, the underlying mechanisms and signals dictating LLPC precursor vs SLPC generation within GCs and factors that can modulate this process remain largely unknown (46). An understanding of the duration of GC reactions needed to drive LLPC generation and clonal dynamics of LLPC clones is also currently lacking in the field (6).

The answering of these questions within the LLPC field has been, in part, impeded by technological limitations which prevented the deep genomic characterization of antigen-specific LLPCs. Recent emerging novel technologies such as nanovials and nanorods (26, 27) and techniques such as Ig trap (29) and TRAPnSeq (28) have enabled genomic characterization of LLPCs at an antigen-specific as well as single-cell level. Although in their infancy, these technologies and techniques show promise as powerful tools to build on current knowledge as well as overcome some of the technical and biological challenges of LLPCs. Combining these technologies and techniques with immunizations within genetic time-stamping mouse models (95, 98) could capture vaccine induced LLPCs, the heterogeneity of this response, and characterize genomic differences in LLPCs to identify a clearer, more unique phenotype for LLPCs. These novel technologies and techniques can further elucidate isotype-specific PC heterogeneity (94) which in the context of vaccination, would be particularly powerful in understanding how to drive IgA LLPCs or IgE LLPCs to provide durable protect against mucosal pathogens (167) or helminths (168) respectively. This level of LLPC profiling can also to be used to enable identification of novel molecular pathways dictating LLPC fate decision making which can be targeted in vaccine design to strategically induce durable immunity.

To advance the understanding of LLPC biology and the development of durable vaccines, there needs to be continued investment in emerging novel technologies that overcome many of the barriers associated with the study of LLPCs. Although mouse models have been instrumental in advancing the current understanding of LLPCs, these models are not fully translatable and therefore necessitate an increased investment in studying LLPCs in NHP models or in human subjects. These investments will be critical to understanding of mechanisms underlying human LLPC generation and with this understanding, can enable the strategic design of vaccines that drive durable, protective immunity.
